# Cation isotopes trace chemical weathering

**DOI:** 10.1016/j.fmre.2023.12.005

**Published:** 2024-01-10

**Authors:** Long-Fei Gou, Fang Huang, Shouye Yang, Gangjian Wei, Zhi-Qi Zhao, Zhangdong Jin

**Affiliations:** a Department of Geography, Chang'an University, Xi'an 710054, China; b SKLLQG, Institute of Earth Environment, Chinese Academy of Sciences, Xi'an 710061, China; c Key Laboratory of Crust-Mantle Materials and Environments, Chinese Academy of Sciences, University of Science and Technology of China, Hefei 230026, China; d State Key Laboratory of Marine Geology, Tongji University, Shanghai 200092, China; e State Key Laboratory of Isotope Geochemistry, Guangzhou Institute of Geochemistry, Chinese Academy of Sciences, Guangzhou 510640, China; f Institute of Global Environmental Change, Xi'an Jiaotong University, Xi'an 710049, China

**Keywords:** Chemical weathering, Li isotopes, Mg isotopes, K isotopes, Ca isotopes, Rb isotopes, Stable Sr isotopes, Ba isotopes

## Abstract

Chemical weathering of rocks and minerals alters the geochemical compositions of the lithosphere, hydrosphere, and atmosphere over time, regulating Earth's surface temperature by consuming atmospheric CO_2_ so as to sustain our habitable planet. As the most mobile species in the processes of chemical weathering, cations are thought to be robust geochemical tracers of chemical weathering. Over the past decades, numerous tracers have been proposed for monitoring chemical weathering, mainly focusing on the contents and ratios of cations. Because of the difference from the properties of cations, information that they provide on chemical weathering over different timescales can be inconsistent or even false, such that some avenues of research have reached an impasse. By virtue of the identical properties of isotopes of the same element and the high-dimensional information that they carry, the stable isotopes of cations have been employed to objectively trace chemical weathering processes, which has become a rapidly developing direction of chemical weathering research. In this review, we summarize the progress made in tracing chemical weathering via the stable cation isotopes (δ^7^Li, δ^26^Mg, δ^41^K, δ^44/40^Ca, δ^87/85^Rb, δ^88/86^Sr, and δ^138/134^Ba) and point out the development trends and persisting problems. After considering the virtues and deficiencies of various cation isotopes, we recommend the combination of multiple cation isotopes that complement and support one another as the future direction to obtain the reliable information on each process of chemical weathering. This should provide the most effective method for objectively tracing chemical weathering, thereby deepening our understanding of the regulatory mechanisms influencing the habitable surficial temperature.

## 1 Introduction

Chemical weathering is the processes whereby crustal minerals are hydrolyzed (and possibly oxidized) by soil solutions and, therefore, is the primary mechanism by which streams, rivers, and ultimately the oceans acquire dissolved salts [1]. It alters the geochemical compositions of the lithosphere, hydrosphere, and atmosphere, regulates global nutrient and carbon cycles, and thus affects life evolution as well as climate change through atmospheric CO_2_ consumption, balancing the degassing of the Earth's interior over a million-year timescale. In this way, chemical weathering profoundly sustains the habitability of the Earth (e.g. [2]). In particular, silicate weathering is considered to be the most important negative feedback mechanism for sustaining the habitable temperature over geological time scales [3,4], whereas carbonate weathering is thought to modulate the global carbon cycle and climate change over shorter timescales (tens of thousands of years) [5,6]. Therefore, how to effectively trace the chemical weathering processes is one of the most important topics in Earth science.

Cation loss from rocks/minerals is the most important process of chemical weathering. Among petrogenetic elements, Li, Na, K, Mg, Sr, Ca, and Ba are the most mobile cations in fluids. The contents and ratios of these cations are therefore often employed to reflect rates, processes, and fluxes of chemical weathering [3,4,7,8], such that they are also known as “geochemical tracers” (e.g. [9,10]). Based on these tracers, several hypotheses relating to chemical weathering have been proposed [3,11,12]. However, because these tracers may be affected by various factors (e.g., bedrock compositions, sedimentation processes, and anthropogenic sources), they are subject to many different influences, resulting in large uncertainties, possible overestimation, paradoxes, and ambiguities, hampering valid understanding of chemical weathering (e.g. [13,14]). New robust geochemical tracers are urgently needed for chemical weathering research.

In the last decade, based on the rapid development of multi-collector inductively coupled plasma mass spectrometry (MC-ICP-MS), various cation isotopes have become determinable and have been employed to trace the flux, rate, and intensity of weathering of silicates and carbonates. The high-dimensional information carried by cation isotopes is expected to quantify the weathering rate, intensity, process, and flux, providing a new tool and fresh insights into chemical weathering. Among these cation isotopes, each isotopic system has its own advantages and disadvantages, such that it might provide more information about a certain aspect of chemical weathering.

In this review, we clarify the principle of using cation isotopes as tracers of chemical weathering, summarize the latest progress, and point out development trends and persisting problems relating to the use of cation isotopic tracers for this purpose. Finally, we propose that chemical weathering is best tracked by applying multiple geochemical tracers and pursuing the mutual corroboration.

## 2 Principles of tracing chemical weathering by cation isotopes

The earth is a rocky planet, and the rocks/minerals exposed on its surface mainly include magmatic, metamorphic, and sedimentary rocks, all of which are in close contact with the hydrosphere, the atmosphere, and the biosphere. Chemical weathering can be expressed as the reactions between rocks/minerals and the ubiquitous water (H_2_O) on the Earth's surface, oxygen (O_2_) and carbon dioxide (CO_2_) in the atmosphere [15]:

(1) (Ca/Mg)SiO_3_ + 2CO_2_ + 3H_2_O = (Ca/Mg)^2+^ + H_4_SiO_4_ + 2HCO_3_^−^

(2) CO_2_ + H_2_O + (Ca/Mg)CO_3_ ⇌ (Ca/Mg)^2+^ + 2HCO_3_^−^

**Fig. 1:**
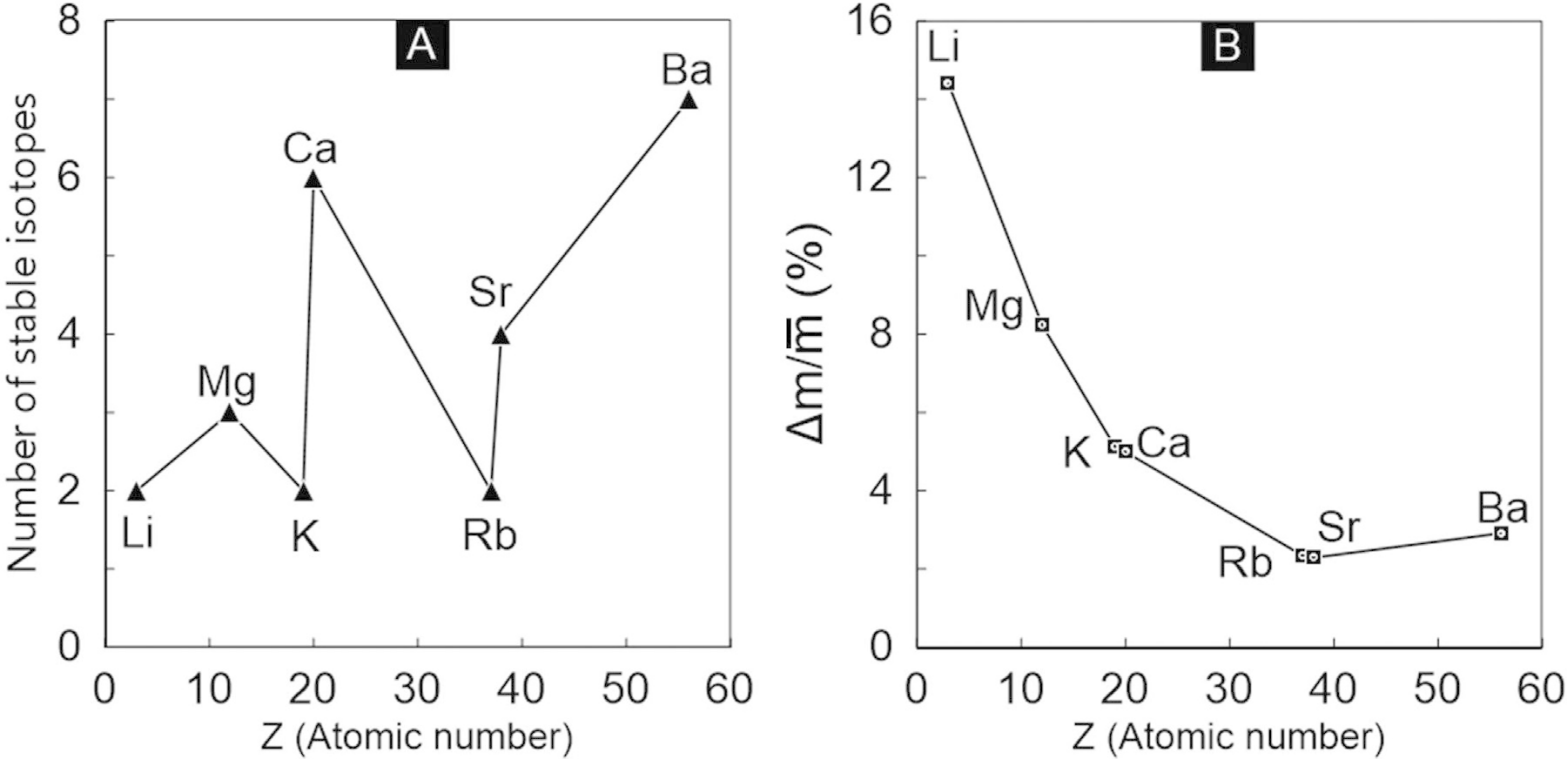
(a) Atomic number versus number of stable isotopes of cations; (b) Difference between the atomic number and relative atomic mass of cations. Note that strictly speaking, rubidium (Rb) has only one stable isotope (^86^Rb), but because of very long half-life of ^87^Rb (6.1 × 10^10^ a), ^87^Rb is considered as “stable” [129].

Among the rocks involved in chemical weathering on the Earth's surface, most sedimentary rocks and sediments are mainly chemically weathered and recycled. Weathering of sedimentary rocks and sediments has consumed very limited atmospheric CO_2_ over the Earth's history [15,16], even though they cover *ca.* 75% of the terrestrial area and nearly 100% of the seafloor [17], besides pyroclastic rocks. In contrast, carbonate weathering serves as an important carbon sink over a millennial timescale [5,6,18]. Unlike sedimentary rocks/sediments, metamorphic and magmatic rocks are formed in the inner Earth, under high-temperature and high-pressure conditions (relative to the surface, 1.01 Kpa, 273 K), making them unstable and easily weathered under surficial conditions. Thus, magmatic and metamorphic rocks are the main reactants, not only releasing nutrients, but also consuming atmospheric CO_2_ over long timescales. In particular, abundant Ca and Mg in mafic/ultramafic magmatic rocks can sequester atmospheric CO_2_ in large quantities and convert it into carbonates as a major part of the sedimentary lithosphere on a million-year time scale [Eqs. 1 and 2]. During the process of chemical weathering, Li, Na, K, Rb, Mg, Ca, Sr, and Ba in magmatic and metamorphic rocks are the most mobile elements (Fig. 1), whereas their structural elements (e.g., Si, Al) and insoluble elements (e.g., Ni, Fe, Ti) tend to remain in the regolith [5,16,19]. Therefore, those mobile elements are the most easily monitored cations in fluids on the continents for quantifying chemical weathering flux, rate, and intensity.

Because of the strong solubility and mobility of cations, chemical weathering flux, rate, and intensity have mainly been quantified based on eight major ions of riverine solutes (K^+^, Ca^2+^, Na^+^, Mg^2+^, Cl^−^, SO_4_^2−^, CO_3_^2−^, and HCO_3_^−^) (e.g. [8,16,19,20]). However, silicate weathering is a slow and long-term process, making it impossible to quantitatively reconstruct chemical weathering flux, rate, and intensity over the geohistorical periods based on the instantaneous riverine solutes. Therefore, the contents and ratios of cation species in archives (e.g., regolith, sedimentary sequences, borehole, etc.) have been served to reflect chemical weathering, with proxies including Rb/Sr, Li/Ba, Mg/Ca, CIA (chemical index of alteration), CIW (chemical index of weathering), and WIP (weathering index of Parker) [9,10]. In fact, the contents and ratios of cation elements can be interpreted with multiple solutions, such that their implications for chemical weathering are rendered uncertain. This is because the contents and ratios of cation elements will vary with (1) different lithological mixtures during chemical weathering in watersheds/profiles/sediments (e.g. [19]); (2) differences in solubility and mobility among the cation elements during the transportation and precipitation processes [7]; (3) the meeting of geochemical barriers during transportation; for example, after flowing through fluids rich in SO_4_^2−^, the mobility of Ba and Ca would be greatly reduced [21], which would invalidate any traceability of the ratios and combination of cation elements such as Li/Ba, Ba/Ca, CIA, and WIP [22]; and (4) anthropogenic input. As a result, how to trace chemical weathering is effectively central to the famous debates between “tectonic uplift-weathering” and “thermostat" hypotheses [2,3,11,12], and latest hypotheses such as island arc collision [23,24]. Therefore, there is an urgent need for quantifiable, repeatable, and verifiable tracers for expediting chemical weathering research for corroboration of the above hypotheses.

There is no difference in geochemical properties among isotopes of the same element, such that cation isotopes can faithfully record process information during chemical weathering [25,26]. The fractionation direction of isotopic systems in fluids is always associated with chemical weathering and subsequent processes. Under this principle, scientists have begun to shed light on weathering processes based on cation isotopes, along with the development of MC-ICP-MS. During recent decades, cation isotopes have been widely applied for tracing chemical weathering. Currently, cation isotopes (δ^7^Li, δ^26^Mg, δ^41^K, δ^44/40^Ca, δ^88/86^Sr, δ^87/85^Rb, and δ^138/134^Ba) have all been determined by MC-ICP-MS with high-precision, thus laying the foundation for their use as tracers of chemical weathering. In this review, we summarize progress made in the use of stable cation isotopes for tracing chemical weathering and then point out their development trends and persisting problems. Among other cations, sodium (Na) and cesium (Cs) have only one stable isotope, while francium (Fr) and radium (Ra) have no stable isotopes, and hence are excluded from this review (Fig. 1).

## 3 Status and problems associated with using cation isotopes to trace chemical weathering

Due to the slight property differences among each cation element, each isotope system is destined to be good at tracing a certain aspect of chemical weathering. The ionic radii of Li, K, Ba, and Rb do not match those of Ca and Mg, and hence the contents of Li, K, Ba, and Rb in carbonate rocks are very low. As a result, they are excellent tracers for silicate weathering. Meanwhile, Ca and Mg are major components of both carbonates and silicates, and hence their isotopes can be used to trace chemical weathering of both carbonates and silicates at the same time. In the following, we review the status and problems associated with each cation isotope system for tracing chemical weathering individually.

### 3.1 Li isotopes

Lithium (Li) has two stable isotopes, ^6^Li and ^7^Li, and the international reference material is LSVEC. The relative mass difference between ^6^Li and ^7^Li (∼16.7%) is the largest of all stable metal isotopes, and its fractionation during chemical weathering can reach 110% (e.g. [27], [28], [29], [30], [31], [32], [33], [34], [35]). Li isotopes are considered as the most promising tracer of silicate weathering and have been extensively investigated recently. This is due to several advantages: (1) As a moderately incompatible element, during the crust-mantle differentiation process, Li enters the rocks in the late stage of crystalline differentiation, leading to much higher Li contents (μg/g level) in silicates than those in other reservoirs [36,37]; (2) Li is repelled by carbonates (ng/g level), so that Li isotopes are only sensitive to silicate weathering [27,28,34,38]; (3) As the lightest metallic element, the large fractionation of Li isotopes is convenient for delineating details of the silicate weathering process [30,39,40]; (4) Li isotopes are essentially not involved in biogeochemical processes; and (5) Li has only +1 electron valence and so is free from redox processes [41]. Due to these features, Li isotopes have been widely used for tracing silicate weathering [28,29,[31], [32], [33], [34], [35],^40^].

There are two main problems associated with Li isotopes for tracing silicate weathering. Firstly, the Li derived from evaporites and hydrothermal inputs needs to be quantified. It has been reported that dissolved Li in watersheds sourced from evaporites can account for 25% or more of the total riverine Li [30,42,43]. Li has also been reported to appear in hydrothermal inputs [44], [45], [46], [47], which fundamentally undermines the use of Li isotopes to trace silicate weathering. Both inputs warrant further quantification of the sources and contributors of dissolved Li globally. Secondly, the control factors of Li isotopic fractionation and its fractionation coefficients are highly controversial. Reported control factors of Li isotopes mainly include: (1) the ratio of primary mineral dissolution to secondary neoformation minerals, which may be expressed as the proportion of Li adsorbed and/or incorporated into solids during chemical weathering [27,34,44,[48], [49], [50], [51], [52]]; (2) temperature dependency on continental scale [43,53]; (3) fluid residence time or water/rock interaction time [40,[54], [55], [56]]; (4) variable Li fractionation coefficients related to the type and proportion of secondary minerals [30,57]; and (5) evapotranspiration, which enriches Li, but with the removal of ^6^Li [58]. Of these, the adsorption rate and capacity of clay minerals and the formation of secondary minerals are currently regarded as the most significant control factors of Li isotopic fractionation [29,32,38,52,59]. As a result, for the application of Li isotopes for silicate weathering reconstruction, clays with appropriate particle size should be selected [31,60]. Overall, the consensus is that Li isotopes are generally a good tracer of silicate weathering on the geological time scale [28,31,34,49].

### 3.2 Mg isotopes

**Fig. 2:**
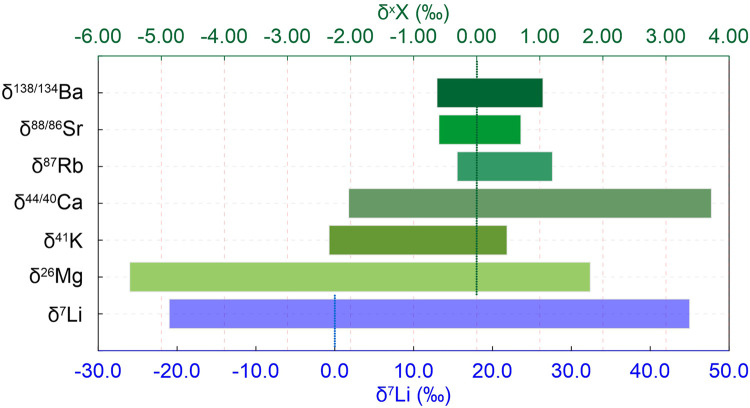
**Variation ranges of stable isotopes of cations.** Among them, Li isotope data are from [34] with an independent bottom x-axis due to its large variation, Mg isotope data from [67], K isotope data from [99], Ca isotope data from [115], Rb isotope data from [129,131], Sr isotope data from [95], and Ba isotope data from [152].

There are three stable isotopes of magnesium (^24^Mg, ^25^Mg and ^26^Mg). Since the homogeneous international reference DSM-3 was successfully developed [61], Mg isotopes have been widely applied in low-temperature geochemistry. The relative mass difference of about 8% between ^24^Mg and ^26^Mg [62], the stable +2 valence free from redox changes, and the prevalence in both silicates and carbonates endorse Mg isotopes with the ability to trace chemical weathering of carbonates and silicates at the same time. On the one hand, there are distinct Mg isotopic compositions of carbonates (−5.57‰ to −0.38‰) and silicates (−0.77‰ to +1.81‰; Fig. 2) [63], [64], [65]. On the other hand, Mg isotopes fractionate significantly during primary mineral dissolution, secondary mineral neoformation, ion exchange, adsorption, and precipitation processes [66], [67], [68], [69]. Thus, Mg isotopes carry valuable information on both sources and processes of chemical weathering [67,70,71].

The main factors controlling Mg isotopic fractionation include (1) Preferred incorporation into the crystal lattice during the formation of secondary clay minerals [72], [73], [74], [75]; (2) Light Mg preferred by the lattice during the formation of carbonates [76,77]; (3) Preferential adsorption and desorption of heavy Mg isotopes by secondary minerals [66,[78], [79], [80]]; (4) Light Mg isotope release in the early stages of silicate weathering [81,82]; and (5) Mixing of materials sourced from solids with different δ^26^Mg values [69,81,83].

There are three challenges associated with the use of Mg isotopes for tracing chemical weathering. (1) Mg is an important nutrient of life, and the extent of the vital effect on Mg isotopes is still subject to debate [82,84,85]. (2) Whether the Mg from different lithologies (with distinct δ^26^Mg values) determines the signal of Mg isotopes in rivers, which in turn can be extended to whether Mg isotopes are sensitive to climatic forcing [86,87]. (3) How to distinguish the signal of chemical weathering from Mg isotopes preserved in various geological archives [88], including marine sediments [88,89], loess-paleosol sequences [33,87], and speleothems [76,77]. Despite the controversy over Mg isotopes, Mg is a major element (strong resistibility to contamination), so that aerosol and dust deposition, groundwater, atmospheric precipitation, and glaciers should have negligible effect on Mg flux in large watersheds [81,90]. Nevertheless, because the chemical weathering of carbonate rocks is one to two orders of magnitude faster than that of silicates [91], the Mg isotopic signal in the watershed is mainly attributable to carbonate weathering. Recently, we proposed that the riverine δ^26^Mg value might be used as an index for the weathering intensity of carbonates in global large river basins [69].

### 3.3 K isotopes

As a moderately incompatible element, potassium (K) has two stable isotopes (^39^K and ^41^K), with SRM3141a as the reference material for K isotopes [92], [93], [94], [95]. With the development of measurement techniques for K isotopes [92], they have started to be used as a new tracer of silicate weathering intensity. Similar to Li, during the mantle differentiation process, K enters the rocks in the late stage of crystal differentiation, so that the K content of the continental crust is much higher than that of the mantle. This makes it convenient to use K isotopes to trace chemical weathering [36,37]. K is mainly contained in silicates (% level) but occurs at very low level in carbonates (μg/g), so that K isotopes are only sensitive to silicate weathering [95], [96], [97], [98]. Their utility stems from the following features: (1) K isotopes are rarely involved in the vital effect [99]; (2) K has only +1 valence and is not subject to redox variation [41]; and (3) K isotopes reach equilibrium quickly during dissolution, with no significant K isotopic fractionation occurring at the basin scale [100,101].

The average δ^41^K composition of the continental crust is about −0.45‰ [102], whereas the ocean has a homogeneous δ^41^K of about +0.12‰ [99,103]. As a result, the riverine δ^41^K values lie between these values. Fractionation of K isotopes is observed at the initial stage of the dissolution process, but quickly reaches to a balanced state. During the migration stage, light K isotopes preferentially enter secondary minerals or are adsorbed by solids, resulting in river and ocean K^+^ being discernibly isotopically heavier [[95], [96], [97],[100], [101], [102],^104^].

The use of K isotopes for tracing silicate weathering is still in its infancy, and some issues need to be resolved: (1) The conclusion that K isotopes enter the secondary mineral lattices during the migration process in the watershed remains speculation [98,105]; (2) Whether marine K isotope archives record temperature control or a vital effect remains uncertain [106,107], despite this being the basis for the application of K isotopes for weathering. Furthermore, according to the results of mass balance calculation of the saprolite, a range of the K isotope fractionation between saprolite and fluid from −0.08‰ to −0.4‰ [95], such that the K isotopic difference between the crust and the ocean appears to be a result of a “reverse weathering” process [108,109]. This warrants further investigations.

### 3.4 Ca isotopes

Calcium has six stable isotopes (^40^Ca, ^42^Ca, ^43^Ca, ^44^Ca, ^46^Ca, and ^48^Ca), and the international reference material is SRM915a. Ca isotopic composition is generally expressed as δ^44/40^Ca or δ^44/42^Ca and is prone to fractionate during migration in surficial geochemical reservoirs (hydrosphere and biosphere). As the fifth most abundant element in the earth's crust and the most abundant alkaline earth metal, Ca isotopes offer traceability of both silicate and carbonate weathering, and thus the global carbon cycle. Indeed, Ca isotopes can link the lithosphere, hydrosphere, biosphere, and atmosphere (e.g., [110], [111], [112]). Due to their insensitivity to redox changes, Ca isotopes have been applied to reconstruct Ca concentrations in paleo-oceans, which are mainly determined by continental weathering, marine carbonate balance, and the pH of geohistorical oceans.

In terms of tracing chemical weathering, the main sources of Ca^2+^ are silicates and carbonates. Thus, the dissolved Ca isotopic ratio was previously thought to directly record the ratio of silicate to carbonate weathering [112,113]. However, it now appears that there are challenges in quantifying Ca isotopic fractionation and conservative mixing. The processes proposed to fractionate Ca isotopes include: (1) Preferential precipitation of heavy Ca isotopes, which may be closely related to the chemical compositions of the accompanying elements, mineral phase, fluids, and physicochemical conditions (e.g., saturation index, stoichiometry, cationic coordination) during precipitation [72,[114], [115], [116], [117]]; (2) The formation and ion exchange of clay minerals [118,119]; 3) Bio-preferred absorption of light Ca isotopes [120,121], and/or 4) Combination of the above processes [122,123].

Similar to Mg isotopes, there are three problems associated with the use of Ca isotopes for tracing chemical weathering of silicate and carbonates. Firstly, it is widely accepted that Ca is inextricably involved in biological processes, such that the research on the use of Ca isotopes for tracing chemical weathering has been limited, being restricted to a few biologically inactive alpine regions. How to evaluate the contribution of Ca fractionation from biological process remains a major challenge. Secondly, the Ca isotope distributions in the ocean are the net result of continental weathering, ocean carbonate balance, and the pH of paleo-oceans. Although the pH of the paleo-ocean can be reconstructed [124], Ca isotopes are obviously affected by continental weathering and ocean carbonate balance processes. Finally, Ca sourced from terrestrial carbonate rocks is modified by ion exchange [20], making it difficult to distinguish the contributions of various inputs.

Overall, it may be difficult to directly trace chemical weathering with Ca isotopes, but they have been widely used to reveal the mechanisms and processes of biological processes [121,125,126]. On the other hand, Ca is the key element of biological skeletons, and organisms preferentially take up light Ca isotopes from the environment. If there were a way to clarify the uptake pathways of biological Ca (metabolic processes), Ca isotopes could become a promising and powerful tool for tracing biological weathering of the continents, which would supplement our poor knowledge on biological weathering [6,91,127].

### 3.5 Rb isotopes

Rb has two isotopes, ^87^Rb and ^85^Rb, with SRM984 as the international reference material. Of these, ^85^Rb is a stable isotope, while ^87^Rb is actually a radioactive isotope that decays to ^87^Sr through β emission. This is one of the most important dating systems. Interestingly, the decay period is very long, reaching 49.76 billion years [128], such that it can be considered as “stable” on the timescale of chemical weathering [129]. Measurement techniques for Rb isotopes are being developed [129], [130], [131].

Because Rb is a lithophilic, fluid-active, and highly incompatible element [37], it enriches in the late magmatic period and is therefore more abundant in the crust than in the mantle [36]. Although the δ^87/85^Rb of the continental crust (−0.14‰ ± 0.01‰) is consistent with that of the mantle (−0.13‰ ± 0.01‰ [131]), Rb isotopes theoretically permit the unique traceability of silicate weathering. This is because adsorption and desorption are the main reasons for the change of its isotopic composition during chemical weathering [129]. To date, however, δ^87/85^Rb data on chemical weathering have been limited, and Rb isotope behavior in the weathering process remains unclear. Since Rb isotopic fractionation appears to be a result of reaction with solids, we surmise that δ^87/85^Rb should be a rather sensitive tracer for silicate weathering intensity. Indeed, more research on watersheds, as well as laboratory and simulation studies, to delineate Rb isotopic fractionation direction and degree during dissolution, precipitation, and adsorption/incorporation processes is urgently needed to fully understand the behavior of Rb isotopes during chemical weathering.

### 3.6 Stable Sr isotopes

As mentioned above, ^87^Rb radioactive decay results in the formation of ^87^Sr, a process that is not only useful for dating, but also for taking ^87^Sr/^86^Sr to quantify the relative contributions of carbonate and silicate dissolution [25]. Recently, however, it has been recognized that the input of metamorphosed carbonate dissolution is difficult to quantify [11,132], and so taking ^87^Sr/^86^Sr as a measure of weathering is still a matter of debate. In order to better constrain the contributions from different rocks and processes of chemical weathering, stable Sr isotopes ratio (δ^88/86^Sr) has been considered for tracing chemical weathering [133], [134], [135], [136], [137]. The accuracy of stable Sr isotopic determination reached 0.03‰, with a δ^88/86^Sr range of −3.65‰ to +1.68‰ in different geological reservoirs (Fig. 2) [134].

Differential dissolution of primary minerals, adsorption and/or entry of secondary minerals, precipitation of carbonates, and even biological processes have been reported as routes that increase the δ^88/86^Sr of fluids [133], but decrease δ^88/86^Sr of the solid phase, showing that these stable Sr isotopes are significantly fractionated during chemical weathering. Fluvial sediment δ^88/86^Sr decreases with increasing weathering intensity, further proving its potential to trace such parameters in watersheds [134,137].

The main problems that hamper the use of stable Sr isotopes to trace chemical weathering are as follows. (1) The extent to which biological effects influence Sr isotopes remains controversial [135,136]. It has been proposed that stable Sr is almost exclusively related to biological processes, but Su et al. (2021) suggested that the chemical weathering process controls stable Sr isotopes [137]. Such controversy seems to be related to the scales and locations selected for scientific purposes. (2) The direction of stable Sr isotope fractionation is also controversial. Han and Eisenhauer (2021) demonstrated that leaves prefer heavy Sr isotopes [136], whereas Guibourdenche et al. (2020) proposed a preference for lighter one [133]. Obviously, more investigations are needed to elucidate the mechanism of stable Sr isotopic fractionation. If the biological disturbance can be qualified, considering the much higher weathering rate of carbonates than silicates, despite the low content of Sr in carbonates, stable Sr isotopes may sensitively reflect both carbonate and silicate weathering. Evidently, however, further targeted investigation is required.

### 3.7 Ba isotopes

Ba is the heaviest stable metal cation considered here and has seven stable isotopes (^130^Ba, ^132^Ba, ^134^Ba, ^135^Ba, ^136^Ba, ^137^Ba, and ^138^Ba), with SRM3104a as the international reference material. Ba isotopes have been used to trace the material cycle between the mantle and the crust [138], as well as the biological Ba cycle in the marine realm [139]. Cao et al. (2016) and Hsieh and Henderson (2017) have reported light riverine Ba relative to seawater [140,141], which can be attributed to the preferential adsorption of light Ba isotopes into (or onto) marine Ba-bearing minerals, making the fluids Ba isotopically heavier (Fig. 2). Due to the nutrient-like behavior of Ba^2+^, Bridgestock et al. (2019) proposed the use of Ba isotopes as a tracer of paleo-productivity [142].

In terms of Ba isotopes tracing chemical weathering, data are still very limited. The reported Ba isotopic fractionation process during chemical weathering is adsorption and desorption [143], [144], [145]. However, Charbonnier et al. (2020) suggested that Ba isotopes are biologically controlled in the Amazon River [146], although Ba^2+^ is commonly regarded as biotoxic [147,148]. Furthermore, Charbonnier et al. (2022) recently proposed that the mixing process of shale and silicates controls the dissolved Ba isotopic composition in the Mackenzie watershed [149].

There is a preliminary consensus that, in the marine realm, Ba isotopes may be useful to trace marine primary productivity [142,150]. Considering the nutrient-like behavior of Ba^2+^, it may be feasible to use Ba to trace the nutrient release process of continental weathering [142,151], alleviating the current lack of understanding of this issue [152]. However, the control factors of Ba isotopic fractionation are controversial, especially due to the lack of self-consistency and the insufficient data. Poor knowledge of Ba isotopic behavior on the continents hinders our understanding of the geochemical cycle of Ba on the surface and thus limits the application of Ba isotopes for tracing chemical weathering.

In summary, the cation isotopes of Mg, K, and Ba fractionate during primary mineral dissolution, whereas those of Li, Ca, and Sr do not (Table 1). Currently, whether Rb isotopes fractionate during primary mineral dissolution is unclear. As a result, Mg, K, and Ba isotopes should be subject to maximum fractionation in regions of rapid erosion regions, potentially allowing for probing the rate of chemical weathering [69,105,144]. Except for radiogenic Sr isotopes, cation isotopes are fractionated during solid scavenging, including adsorption and incorporation into solids that are difficult to distinguish in nature. Among them, light isotopes of Li, K, Rb, Sr, and Ba tend to enter secondary aluminosilicate minerals preferentially, as do heavy isotopes of both Mg and Ca generally, though a preference for light Mg and Ca isotopes has also been reported (Table 1). From this perspective, the combination of one or two of the Li, K, Rb, Sr, and Ba isotopes with either Mg or Ca isotopes may give an indication of the significance of secondary aluminosilicate neoformation in watersheds [81,153]. On the other hand, carbonate dissolution generally does not fractionate cation isotopes, but carbonate precipitation prefers light Mg and Ca isotopes. As a result, a combination of Mg and Ca isotopes may provide a means of tracing whether carbonate precipitation is important in a given system [71,154]. During the transportation, dry (wet) atmospheric input will inevitably be incorporated and thereby change the cation isotopic compositions. However, the available data show that the major elements, Mg, K, and Ca isotopes have strong anti-contamination resilience [71]. Instead, the trace elements and their isotopes are often impacted by dry (wet) precipitation, i.e. poor anti-contamination resilience. Hence, correction for atmospheric input is recommended before considering cation isotopes to trace weathering.

**Table 1:** **The cation isotopic preference during various processes of chemical weathering**.

Isotopes Processes	Silicate dissolution	Carbonate dissolution	Evaporite dissolution	Clay incorporation	Clay adsorption	Carbonate precipitation	Biological preference	References
δ^7^Li	−	−	−	*L*	*L*	−	*L*	[27,40,48,53,58]
δ^26^Mg	*L*/*H*	−	−	*L*/*H*	*H*	*L*	*L*	[33,62,66,71,74,75,84]
δ^41^K	*H*	−	−	*L*/*H*	*L*/*H*	−	*L*	[[91], [92], [93],^98^,^101^,^106^]
δ^44/40^Ca	*H*	−	−	*H*	*H*	*L*	*L*/*H*	[111,113,115,118,122,127]
δ^87^Rb	unclear	−	−	unclear	*L*/*H*	unclear	*L*	[129], [130], [131]
δ^88/86^Sr	*H*	*H*?	−	*H*?	*L*	*L*	*L*/*H*	[133], [134], [135], [136], [137]
δ^138/134^Ba	*L*	−	−	*H*	*H*	*L*	*L*	[126,138,141,144]

*H* refers to prefer heavy isotopes; *L* refers to prefer light isotopes; − refers to no preference to isotopes.

It should be kept in mind that there is uncertainty since the reported data are occasionally debated to each other.

**Fig. 3:**
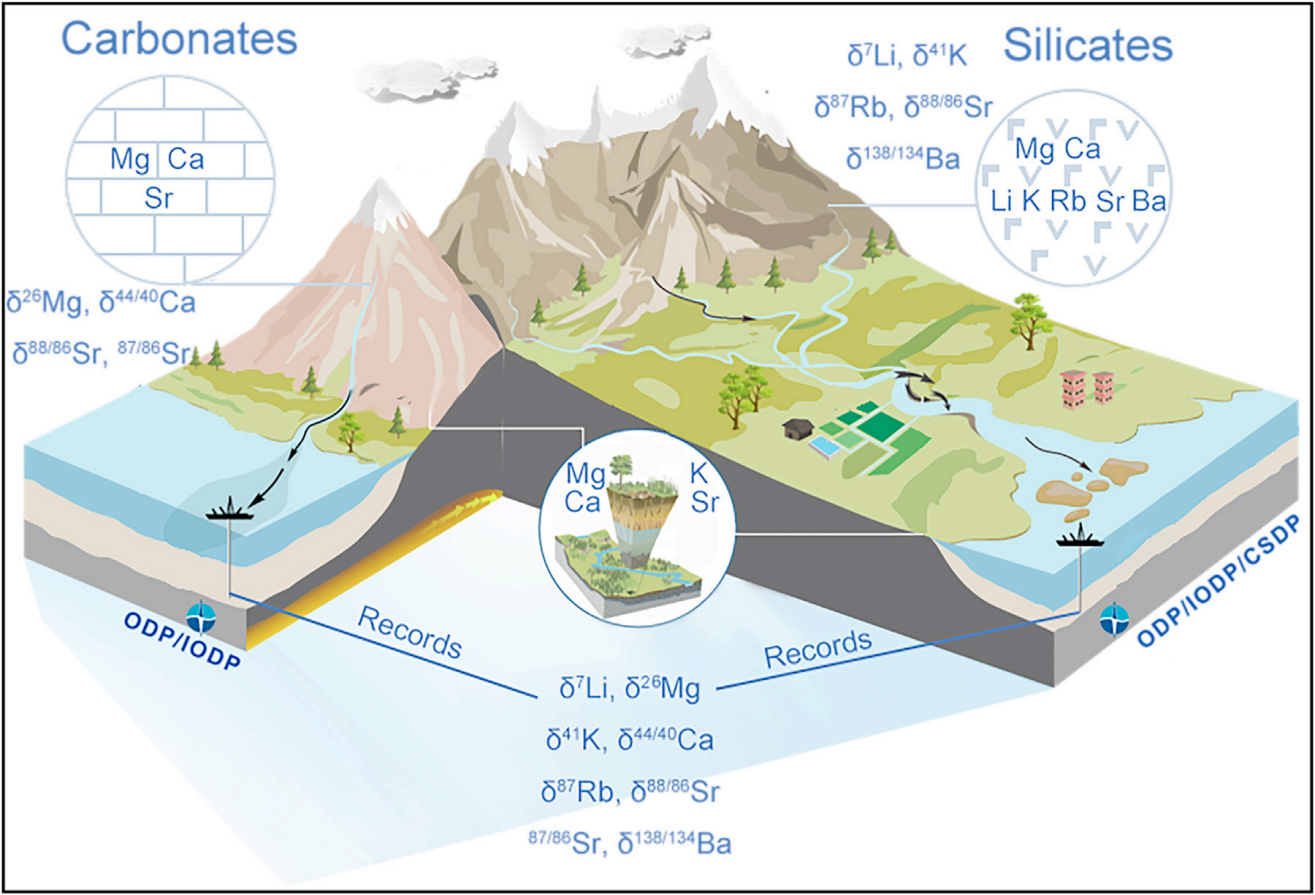
**A cartoon illustrating the processes of dissolution, transportation, removal, and signal preservation of both cation isotopes and chemical weathering of silicates and carbonates at typical settings on Earth's surface**.

It should be kept in mind that each cation isotope has its own deficiencies for tracing chemical weathering, such that the combination of multiple cation isotopes should yield reliable information on the rate, processes, and flux of chemical weathering (Fig. 3). Moreover, all cation isotopes are involved in the vital effect, with Ca and Mg isotopes being most significantly affected by biological processes. Attention needs to be paid to the vital effect when Ca and Mg isotopes are applied to trace geohistorical chemical weathering after the Ediacaran period (life explosion).

## 4 Further understanding chemical weathering based on geochemical tracers

Cation in modern rivers and regoliths derive from atmospheric inputs (including dry and wet precipitation), silicates, carbonates, evaporites, biomass disturbance, and anthropogenic inputs. There was certainly no anthropogenic input in deep time. When we try to understand the surficial temperature change of the habitable Earth on a million-year timescale, Li, K, Rb, radioactive and stable Sr, and Ba isotopes can be consulted to trace silicate weathering, including the part of the silicates derived from dust/wet precipitation [3]. When we focus on the regulation of atmospheric CO_2_ by chemical weathering on a millennial timescale, Mg, Ca, and radioactive and stable Sr are the key isotopes to trace carbonate weathering [5,6].

The sources and fractionation processes of cation isotopes are mutually variable, such that their traceability permits comprehensive understanding of chemical weathering from multiple perspectives (Fig. 3). Among them, Li and K isotopes are expected to be robust tracers of silicate weathering intensity, but both pairs should be applied jointly, since Li isotopes evade the vital effect and K isotopes are not subject to evaporite disturbance [105,155]. For tracing carbonate weathering intensity, Mg and Ca isotopes may be reliable tracers, but the vital effect on both isotopes and their isotopic signal preservation in archives need careful evaluation [120,121]. As Ca is a vital nutrient for life, Ca isotopes fractionate during biological activities. When there is a suitable archive, Ca isotopes may be a promising tracer of biological weathering, with the silicate weathering background being obtained from Li and Mg isotopes [51,69]. Due to the nutrient-like behavior of dissolved Ba, its isotopes are becoming established as a tracer of nutrient supply through chemical weathering from the land to the ocean [142].

In fact, each geochemical tracer has its specific scope of application, including both cation isotopes and elemental ratios. In this context, it is essential to deeply understand the rates, processes, and fluxes of chemical weathering using either cation isotopes or elemental ratios, though the ratios of cation elements can have multiple solutions, as mentioned above. For example, as one of the widely used tracers for chemical weathering, sedimentary Rb/Sr ratio is mainly controlled by Sr from weathering of both silicates and carbonates, but it is easily truncated at low values under strong weathering [156], such that it no longer reflects the real intensity of weathering. The combination of Rb/Sr ratio with Rb isotopes and radioactive and stable Sr isotopes is expected to allow accurate quantification of the carbonate and silicate proportions in chemical weathering. Similarly, CIA can effectively trace weathering intensity in single regolith [9] or sediment sequences with stable sources, but it may contain other information associated with transportation and precipitation processes when applied to various sediments. Analyses of sedimentary Mg and K isotopes might help to understand the impact of transportation and precipitation processes on elements in CIA, and thereby to reflect weathering using CIA and Mg and K isotopes.

Overall, for the complex Earth surface system, geochemical perspectives from multiple cation isotopes with elemental ratios are naturally warranted (Fig. 3). For example, when we explored the seasonally stable Li, Mg, Ba, and radiogenic Sr isotopic variation in the middle Yellow River and in the Buha River within the Lake Qinghai basin, we found that their behaviors reflect different processes involved during chemical weathering. Specifically, Li isotopes showed a temperature dependence; Mg isotopes showed fractionation during mixing, secondary carbonate precipitation, and clay formation; radiogenic Sr appeared as a result of mixing; and Ba isotopes indicated adsorption. Moreover, Mg/Ca and Sr/Ca ratios reflected seasonal variations in carbonate weathering and secondary carbonate precipitation [40,43,71,144].

From a microscopic perspective, cation isotopic fractionation is controlled by (1) the relative amounts of silicate and carbonate dissolution, (2) the rate of adsorption on solids, (3) the neoformation rate of secondary aluminosilicates, and (4) the secondary carbonate precipitation rate. From a macroscopic perspective, tectonics, lithology, geomorphology, and climate determine both chemical weathering and the associated cation isotopic fractionation. There are many variables, and they can superpose on each other. Unfortunately, the existing samples for geochemical tracers were collected spatiotemporally, so they contain information on tectonics, lithology, and geomorphology pertaining to cation isotopes, as well as signals from climate and hydrology. The results represent a complex superposition of various factors and can even yield contradictory conclusions (e.g. [136,137]).

Determining every variable that controls chemical weathering is a powerful way to further investigate the behaviors of cation isotopes during weathering; thereafter, each factor may be quantitatively elucidated by one or more cation isotope, and finally they may be modeled collectively. The following actions are recommended:

(1) By collecting and analyzing high-resolution time series samples at various climatic settings, ideally at small, monolithological watersheds, after the influences of tectonics, lithology, and topography on chemical weathering and cation contents are ruled out, the response of cation isotopes to climate (temperature and precipitation) may be exclusively quantified.

(2) To delineate the roles of tectonics/lithology/topography on cation isotopes, samples pertaining to a specific role should be analyzed and investigated for one or more cation isotopes.

(3) To gauge the impacts of adsorption and incorporation processes on cation isotopic fractionation, analysis of the cation isotopes in various species by stepwise leaching of the target solids combined with laboratory synthesis experiments may serve to quantify the respective fractionation coefficients of cation isotopes.

(4) On the basis of increasing natural observations and data, the basic thermodynamics of isotopes, the first-principles calculations, numerical simulation, and machine learning (such as deep learning, transfer learning) for cations and their isotopes should provide a quantitative understanding of the behavior of cation isotopes and thus the complex processes of chemical weathering based thereon, in particular the coupling of multiple isotopes.

Through the efforts of the above combined actions, parameters may be simulated by models and processed by artificial intelligence (AI) for machine learning, after geohistorical chemical weathering is reconstructed on the basis of cation isotopes. In the near future, the rate, intensity, and flux of chemical weathering in modern and deep time may be quantitatively revealed.

## Declaration of competing interest

The authors declare that they have no conflicts of interest in this work.
